# Best practices when benchmarking CATCH for the design of genome enrichment probes

**DOI:** 10.1093/bioinformatics/btag002

**Published:** 2026-01-13

**Authors:** Hayden C Metsky, Katherine J Siddle, Christian B Matranga, Pardis C Sabeti

**Affiliations:** Broad Institute of MIT and Harvard, Cambridge, MA 02142, United States; Broad Institute of MIT and Harvard, Cambridge, MA 02142, United States; Broad Institute of MIT and Harvard, Cambridge, MA 02142, United States; Broad Institute of MIT and Harvard, Cambridge, MA 02142, United States

We previously described CATCH, a computational method that designs probe sets for enriching genomes from diverse taxa prior to sequencing (Metsky et al. 2019). A recent paper in *Bioinformatics* describes a related method, Syotti, and compares it to CATCH (Alanko et al. 2022). Methodological developments are needed in the field, and we are excited to see a focus on them. Yet, in their benchmarking, Syotti’s authors made five decisions that go counter to a fair comparison and paint a misleading picture of CATCH’s performance. To uphold benchmarking best practices for the field and ensure that future users are not misguided in using CATCH, we describe the five decisions below. Decisions (1)–(3) involve measuring runtime. Decisions (4) and (5) involve analyzing output.

The authors in Alanko et al. (2022) emphasize that CATCH’s runtime is impractical and scales poorly with large input. Under different benchmarking decisions—that, instead, are true to how CATCH is typically run—CATCH is orders of magnitude faster than reported (Fig. 1a). In practice, these decisions can make a difference between several months or several hours of runtime. Three decisions allow for the authors’ conclusions about runtime:

**Figure 1:**
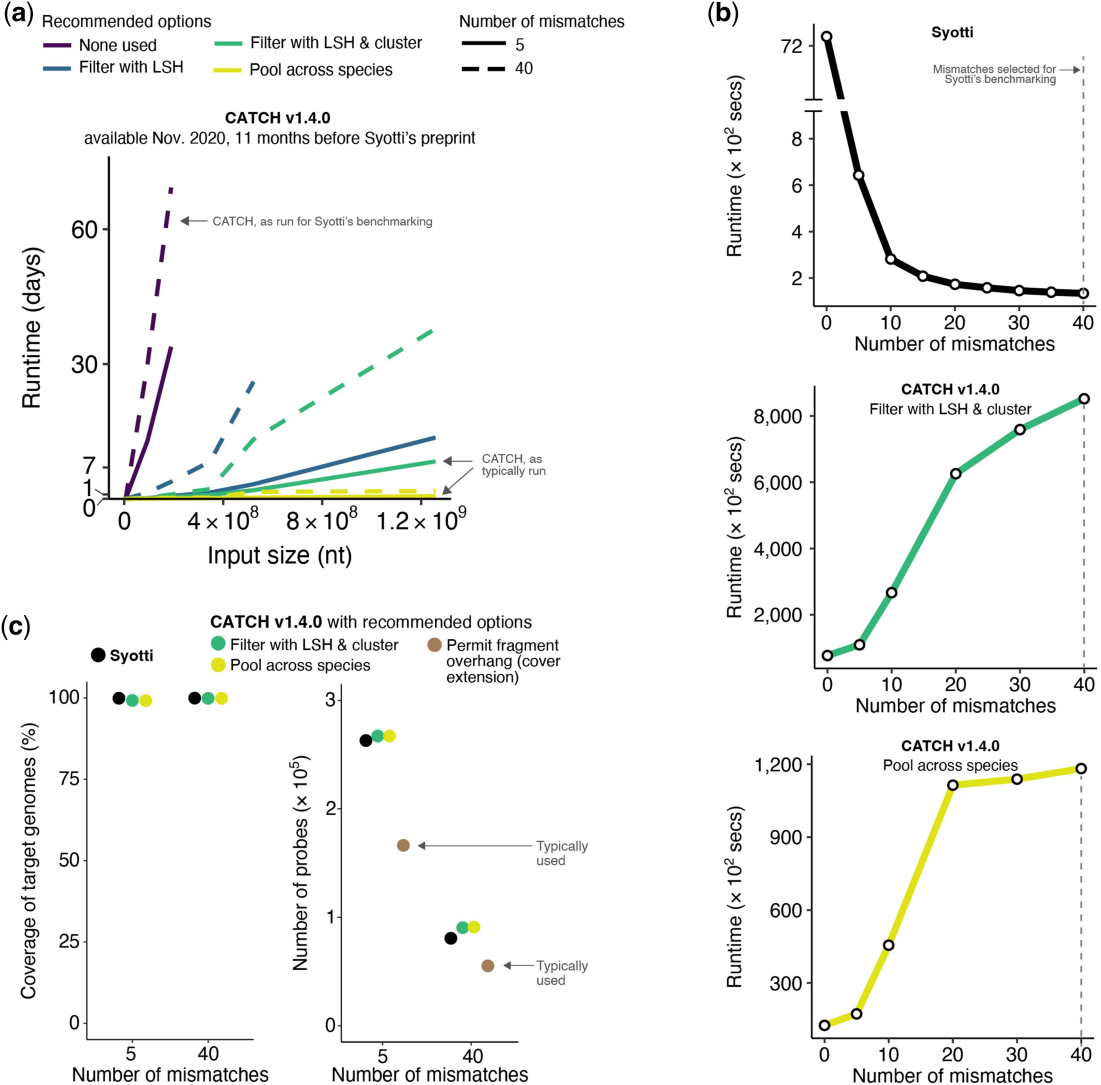
User-specified arguments affect the runtime of CATCH as well as comparisons of coverage and probe counts. (a) The runtime of CATCH v1.4.0—available 11 months before Syotti was described in a preprint—when run with increasingly large input samplings. The input is the viral dataset used in Syotti’s benchmarking (Alanko et al. 2022), which we had previously constructed and made available; it contains 588 viral species. Colors indicate a manner of running CATCH: purple indicates using no recommended options, and blue and green indicate using options recommended in CATCH’s publication, README, and help messages. Yellow indicates using viral species labels provided in the data, by handling species independently and pooling probes across them (pooling has little effect on the number of probes owing to low whole-genome homology across species; **Figure S3**). Solid/dashed line types indicate the probe-target divergence (number of mismatches) tolerated in the design; CATCH exhibits lower runtimes at lower values of this parameter, which contrasts with Syotti’s behavior (see (b)). Syotti’s runtime benchmarking (Alanko et al. 2022) runs CATCH as shown by the dashed purple line, which uses no recommended options and 40 mismatches. The jagged pattern of the lines results in part from following the input sampling strategy used in Syotti’s benchmarking (Alanko et al. 2022), in which entire groups of species are successively introduced into the input. ∼1.26 × 10^9^ nt of input is the full viral dataset; lines that end before that input size represent runs of CATCH that were terminated early owing to their expected runtime. “Filter with LSH” refers to the argument ′--filter-with-lsh-minhash 0.6′; “cluster” refers to the arguments ′--cluster-and-design-separately 0.15 --cluster-from-fragments 50000′; and “Pool across species” refers to leveraging taxon labels to design independently on viral species. (**b)** Runtime of Syotti (top) and CATCH (middle and bottom) when run with different values for the parameter governing the number of mismatches to tolerate between a probe and target, that is, the allowed probe-target divergence. Syotti’s runtime benchmarking (Alanko et al. 2022) sets the value to 40 mismatches for both tools. (c) Left: coverage of targeted genome sequences, as a percentage of all nucleotides, achieved by probe sets designed with Syotti and CATCH, as computed by Syotti’s coverage analyzer. Horizontal axis is the allowed probe-target divergence for probe design, and the divergence is checked when computing coverage. In all cases, coverage is >99%. Right: Number of probes in the output of Syotti and CATCH. Brown indicates a recommended option (′-e 50′), typically set in practice; it models capture by assuming that hybridization captures the portion of a DNA fragment overhanging where the probe hybridizes (i.e. the full fragment), not only the portion of the fragment hybridized to the probe. (Coverage, in the left panel, is not shown using this option because Syotti’s coverage analysis program does not consider fragment overhangs.) In (b) and (c), analyses use an input of 256 viral species (5.22 × 10^8^ nt).

1. The benchmarking disregards CATCH’s options that substantially lower runtime and memory usage on large inputs. Those options are recommended in CATCH’s publication, README, and help messages specifically for that purpose. Turning on those options dramatically decreases CATCH’s runtime and improves its scalability (Fig. 1a and Supplementary Figs S1 and S2). Their trade-off is an increase in the number of probes; since this increase is small (<10% for large input; Supplementary Fig. S3), we often use these recommended options in practice.
2. The benchmarking sets a critical design parameter, the allowed probe-target divergence, to a single unrepresentative value, where Syotti runs relatively fast while CATCH runs relatively slow. The chosen value is higher than the probe-target divergence at which there is a well-established reduction in probe-based enrichment efficiency, and considerably higher than other groups and we have ever allowed in practice, or ever considered allowing. When setting this divergence parameter to lower values that are more relevant in practice, CATCH runs much faster (Fig. 1b); Syotti displays the opposite behavior with this parameter, exhibiting longer runtimes at lower values (Fig. 1b).
3. The benchmarking runs CATCH in a different manner on viruses—the data on which it shows CATCH having the worst performance—than we have described. CATCH benefits from viral species labels that are typically available in datasets, including the one used in Alanko et al. (2022). The benchmarking removes species labels and takes a subroutine intended for one species—which we describe and illustrate in our publication as applied to one species—and applies it to many species at once. That choice dramatically affects CATCH’s runtime (Figure 1 and Supplementary Fig. S1) while making little difference to the output’s optimality (Supplementary Fig. S3).
  The authors in Alanko et al. (2022) report that Syotti provides more favorable output than CATCH. They conclude, erroneously, that Syotti yields (i) more coverage of targeted genomes and (ii) fewer probes than CATCH. Two decisions allow for those conclusions:
4. The benchmarking selects different design parameters for probes designed with CATCH versus Syotti, but evaluates the probes as if designed with Syotti’s parameters. Specifically, it compares CATCH’s probes, designed using relaxed hybridization criteria, to Syotti’s probes, designed using relatively strict criteria. The evaluation decides which genomic regions are covered according to the criteria given to Syotti. Syotti’s probes always meet that criteria, whereas CATCH’s probes, by design, do not—thus, affording Syotti’s probes more genomic coverage. The evaluation, therefore, concludes mistakenly that CATCH yields designs with low coverage, for example, 84% coverage in one comparison (Alanko et al. 2022). If CATCH were given the same parameter values as those given to Syotti—as it could and should have been in such a comparison—the evaluation would find that the methods yield comparable coverage (both >99% coverage; Fig. 1c, left).
5. The benchmarking, when comparing the number of output probes, does not use an option in CATCH that reduces that number by about 30%–80%. We typically use this option in practice, including in Metsky et al. (2019), as do other users of CATCH. With this option, CATCH designs a probe set with 36% fewer probes than Syotti (Fig. 1c, right). We also emphasize that, even without applying this option, Alanko et al. (2022)’s conclusion that Syotti outputs fewer probes than CATCH is well substantiated by only one of the three datasets analyzed.

Supplementary Text provides further details about each of the above decisions and their effects on benchmarking.

Informed by this experience, we expanded CATCH to enhance its usability when it is run in a manner that overlooks or runs counter to our public recommendations. We introduced a new command, in CATCH v1.5.0, that makes our publicly recommended options the default settings, thus providing a command for users with limited familiarity with CATCH (Figure S1). We hope that CATCH—with its 5200 lines of code documentation, 308 automated tests, modular implementation, and automated access to NCBI viral genomic data—can continue to grow while providing reliable output. We also look forward to seeing Syotti and other methods continue to develop, and hope that we can all work to fairly compare our results in the service of advancing science.

## Supplementary Material

btag002_Supplementary_Data

## Data Availability

No new data were generated.

## References

[btag002-B1] Alanko JN , Slizovskiy IB, Lokshtanov D et al Syotti: scalable bait design for DNA enrichment. Bioinformatics 2022;38:i177–i184. 35758776 10.1093/bioinformatics/btac226 PMC9235489

[btag002-B2] Metsky HC , Siddle KJ, Gladden-Young A, et al; Viral Hemorrhagic Fever Consortium. Capturing sequence diversity in metagenomes with comprehensive and scalable probe design. Nature Biotechnology 2019;37:160–8. 10.1038/s41587-018-0006-x PMC6587591 30718881

