# Supplementary material for: Best practices when benchmarking CATCH for the design of genome enrichment probes
Source: Bioinformatics. 2026 Jan 13;42(5):btag002. doi: 10.1093/bioinformatics/btag002 (PMC13171601; doi:10.1093/bioinformatics/btag002)
Supplement: btag002_Supplementary_Data [file btag002_supplementary_data.zip › Supplementary_Text.docx]

# Supplementary Text

This supplementary text provides a detailed description about each of the points in the main text. Details include the particular options and parameters mentioned in the main text, as well as their effects on evaluating CATCH [1].

## Options that improve runtime and memory usage

CATCH offers options, which are unused in [2], to enhance its runtime and memory usage. As our publication's main text explains, "CATCH can use locality-sensitive hashing (LSH), if desired, to reduce the number of candidate probes that are explored, improving runtime and memory usage on especially large numbers of input sequences." Specifically, as CATCH's README notes, the LSH option `--filter-with-lsh-minhash` "can significantly improve runtime and memory requirements when the input is especially large and diverse." Thus, we recommend that users consider the option when CATCH's runtime or memory usage are barriers. A reasonable starting value is `--filter-with-lsh-minhash 0.6`.

The benchmarking in [2] disregards the feature even though it concludes that CATCH exhibits poor runtime and memory usage. [2] does observe that overlooking the option "might in retrospect have had significant impact on [CATCH's] running time." For instance, on the MEGARes dataset, the benchmarking in [2] shows that CATCH exhibits worse than linear (super-linear) runtime behavior as the input grows while its own method exhibits a more desirable linear growth; when using the LSH option, CATCH's performance is linear too on that dataset (R^2^=0.996; Supplementary Figure 2). On the viral dataset, **that option lowers CATCH's runtime by 94.5%** (evaluated at 15.1% of the full dataset^^[[1]](#footnote-2)^^; Figure 1a). Enabling a second runtime-focused option that clusters input sequences—which CATCH's README describes as "another option to improve runtime and memory requirements" (`--cluster-*`)—further reduces runtime. **The combined reduction in runtime is 98.6%** on 15.1% of the data (Figure 1a), with the reduction in runtime becoming more marked as the input grows.

When reporting results, we used `--filter-with-lsh-minhash 0.6` for the LSH option and `--cluster-and-design-separately 0.15 --cluster-from-fragments 50000` for the clustering-based options. The results above derive from using the options with CATCH [v1.4.0](https://github.com/broadinstitute/catch/releases/tag/v1.4.0), which was available in November 2020, 11 months before [2] was posted as a preprint. With CATCH [v1.5.0](https://github.com/broadinstitute/catch/releases/tag/v1.5.0), a more recent version available in September 2022, **the LSH option lowers CATCH’s runtime by 96.2%** and, using the second runtime-focused option noted above, **the combined reduction in runtime is 99.6%** on 15.1% of the data (Supplementary Figure 1; see legend for additional details about benchmarking).

These recommended options have two tradeoffs. They usually yield about 1 to 15% more probes (Supplementary Figure 3). And, when specifying low probe-target divergences (below ~3 mismatches), the LSH option can yield less than perfect target coverage. These tradeoffs might be relevant to some users, but they are not relevant to the benchmarking in [2], which sets a high probe-target divergence (40 mismatches) and highlights runtime results that would have benefitted from the runtime-enhancing options.

## A critical design parameter: the allowed probe-target divergence

The limit on probe-target divergence is an important variable when designing probes, but is not considered in the benchmarking in [2]. Most of the benchmarking sets a 40-mismatch limit on probe-target divergence (33% of the probe length)^^[[2]](#footnote-3)^^. That 33% threshold is much higher than we have tested with CATCH (up to 11% [1]) and that other groups that we are aware of have allowed for comprehensive capture panels (e.g., 10% in [3]). It is also past the limit at which we [1] and others [4,5] have observed robust enrichment in practice; these studies [1,4] describe a marked reduction in enrichment efficiency beyond ~20% divergence. Thus, while 33% divergence might be marketed by a DNA manufacturer as tolerable (see footnote 2), it is not ideal nor representative of typically-used values, and might not even be useful in practice.

Aside from impacting a probe set's utility, the probe-target divergence limit affects runtime. Two critical subroutines in CATCH are optimized for lower, more conservative divergences. Running CATCH to enforce lower divergences—similar to the values that yield high enrichment in practice—reduces CATCH’s runtime considerably (Figure 1a,b and Supplementary Figure 1). Syotti [2] shows the opposite behavior in our testing, exhibiting slower runtimes at those lower, more cautious divergences (Figure 1b). If permitting 0 mismatches on the viral dataset (`--mismatches 0`), for example, **CATCH's runtime decreases by 97.4%** compared to 40 mismatches (15.1% of the full dataset; see footnote 1) with that change alone, whereas Syotti’s runtime increases by 34-fold (full dataset). At 5 mismatches (`--mismatches 5`), **CATCH’s runtime decreases by about 30% to 90%** compared to 40 mismatches, depending on the input size and specified options (Figure 1a). Therefore, the choice to benchmark runtime at just one high and unrepresentative divergence cutoff—where CATCH performs at its worst, and to not consider other values—disadvantages CATCH.

The benchmarking concludes that "Surprisingly, CATCH itself was unable to process … 1% of the full dataset within the time limit of 72 h." Combining just a lower divergence (5 mismatches) with the recommended options described above, CATCH processes 42% (256 species) of the full dataset well within that time limit (47 h with CATCH v1.4.0; 18 h with CATCH v1.5.0). **CATCH v1.4.0 processes 100% of the full dataset in 200 h** for 5 mismatches (909 h for 40 mismatches; Figure 1a); with v1.5.0, it processes 100% of the full dataset in 105 h for 5 mismatches (140 h for 40 mismatches; Supplementary Figure 1). When considering runtime, users should bear in mind this important variable.

## Taxon labels in genomic datasets

Large viral genome datasets—including the dataset used in the benchmarking in [2]—usually organize or label genomes by their species. CATCH takes advantage of viral species labels. They help CATCH further enhance its runtime (species can be handled independently owing to low whole-genome homology) and, more importantly, account for species having different degrees of variation; a primary goal of CATCH is to balance the probe set's composition between the less diverse species having few probes with the more diverse species that might otherwise dominate the probe set. That idea is central to how we built CATCH, described it in our publication [1], and how we have tested and applied it. The practice in [2], by contrast, takes a different approach to running CATCH. It disregards species labels provided by the data and takes a subroutine algorithm intended to be applied to one species—which is described in our publication as being meant for one species and illustrated as being applied to one species (Figure 1a of our publication [1])—and applies it to as many as 588 at once. In practice, designing on independent viral species and pooling across them has minimal impact on the size of the probe sets given low whole-genome homology across species (Supplementary Figure 3).

Evaluated on 15.1% of the full dataset (see footnote 1), the commands used in [2] take 69 days for 40 mismatches or 34 days for 5 mismatches; running CATCH in our described manner, it takes 6 h for 40 mismatches or 2.7 h for 5 mismatches (CATCH v1.4.0, Figure 1a; with CATCH v1.5.0, 5.5 h for 40 mismatches or 1.25 h for 5 mismatches, Supplementary Figure 1). **On the full viral dataset, running CATCH in our described manner, CATCH v1.4.0 processes the full viral dataset in under 15 h for 5 mismatches or under 43 h for 40 mismatches** (Figure 1a); with CATCH v1.5.0, the runtimes are under 7 h for 5 mismatches or under 38 h for 40 mismatches (Supplementary Figure 1). **These runtimes, for 5 mismatches, are about 99.9% faster than runtimes required by the commands used in [2]**, if the runtimes of those commands are extrapolated linearly from 15.1% of the dataset to the full dataset.

The viral dataset is where [2] shows CATCH having the worst performance, but a similar idea applies to non-viral data. Taxon labels are sometimes less obvious albeit still helpful; for instance, with pan-bacterial panels, we have treated different core genes as the "species". The `--cluster-and-design-separately` and `--cluster-from-fragments` arguments in CATCH aim to emulate that process, including for bacteria, by breaking genomes into fragments and designing on independent clusters.

## Genomic coverage analyses and the impact of design parameters

Limits on probe-target divergence impact the analysis of a probe set's genomic coverage. Users should pay attention to the divergence specified during both design and analysis. The benchmarking in [2] demonstrates a pitfall. In a particular analysis, it decides uncovered genomic regions to be ones that are more divergent than 5 mismatches from any probe. For that analysis, the benchmarking designs a probe set with Syotti that allows up to 5 mismatches from genomic targets, and compares that probe set to ones designed by CATCH that allow up to 8 mismatches^^[[3]](#footnote-4)^^. That inconsistency disadvantages CATCH in the comparison; by design, CATCH's probes will not always meet a 5-mismatch threshold whereas Syotti's probes will. This is one reason for the suboptimal coverage that [2] reports for CATCH's designs, such as 84% for one probe set (the next paragraph gives the second reason). The result is the evidence for the conclusion, highlighted in [2]’s abstract, that its method "leaves fewer positions uncovered". When analyzed with criteria that are consistent with design parameters, CATCH's probe sets, like Syotti’s, provide >99% coverage of input sequences (Figure 1c).

A related pitfall can arise during analysis, this one involving the definition of which genomic regions are covered by probes. A probe is typically shorter than the DNA fragment to which it binds, allowing genomic regions to be enriched even if a probe does not directly overlap them. CATCH optionally reflects that property (see Supplementary Figure 1b in CATCH’s publication [1]). Leveraging a fragment's "overhang" immediately surrounding the probe's binding site—CATCH calls it a "cover extension"—helps to reduce the number of probes while making them more robust to variation: CATCH positions probes so that hypervariable regions fall in overhanging, non-binding parts of a fragment. This optional feature offers a practical benefit to CATCH’s designs. We recommend that users bear in mind this feature during benchmarking. The benchmarking in [2] analyzes the coverage of CATCH's viral probe sets when designed to allow for overhangs, yet it does not account for the overhangs when calculating the coverage of those probe sets. That inconsistency disadvantages CATCH in the comparison because the definition of which positions are covered differs between CATCH's design and the analysis; by contrast, the definition is consistent for Syotti. That choice is the second reason for the suboptimal coverage that [2] reports for CATCH. When accounting for overhangs that are a feature of the design, CATCH's probe sets are designed to provide full coverage; alternatively, the probe sets could be designed without allowing overhangs and analyzed without them, in which case we anticipate that they would also demonstrate full coverage.

## Probe set sizes and the impact of design parameters

Although Syotti’s genomic coverage relative to CATCH, as reported in [2], benefits from overhangs in CATCH’s designs, the benchmarking in [2] does not use overhangs in CATCH when evaluating CATCH’s probe set sizes. The benchmarking compares CATCH's 303K probes to its own 189K on a bacterial dataset, thereby concluding in its abstract that Syotti "produces bait sets that are smaller than the ones produced by [CATCH]". (It is worth noting that, as far as we can tell, this conclusion is only well supported by that one dataset: on the other two analyzed datasets (MEGARes and viral), the probe sets have similar sizes.) Leveraging the overhangs strategy usually reduces the number of CATCH's probes by about 30–80% depending on the input and other design parameters; thus, the option affects comparisons of probe set sizes. On the viral dataset analyzed in [2], CATCH designs a probe set with 36% fewer probes than Syotti (at 5 mismatches; at 40 mismatches, 31.5% fewer probes than Syotti; Figure 1c). This option sometimes reduces runtime as well because CATCH can more rapidly cover the most variable regions. We recommend that users pay attention to this feature, via the `-e` argument, during the design and analysis of probe sets.

Reflecting on the use of probe-target divergence and overhang settings in [2], we recommend that benchmarking studies comparing two probe design methods either (a) specify commensurate design decisions between the two methods or (b) reflect any inconsistent design choices in the analysis, rather than use fixed criteria matching its own method's design choices. Doing so can offer more even-handed comparisons.

## Updates to CATCH

We have expanded CATCH to enhance its usability when run in a manner that overlooks publicly recommended options. We introduce a new command, `design_large.py`, available in CATCH v1.5.0. This command accepts fixed parameters on design criteria specified across all taxa, rather than balancing a probe set's composition among taxa using variable criteria as we have previously described. This command enables the LSH option by default as well as the option to cluster input sequences for approximating taxa, a process we have now optimized to use less memory. The command also enables, by default, a previously available option (unused in [2]) that has helped CATCH process bacterial genomes by breaking them into chunks; this capability is further improved in v1.5.1. Together, the command offers users, with minimal familiarity with CATCH, more efficient handling of large numbers of diverse viral taxa or bacterial genomes without needing taxon labels that divide the input. Implementation details are in [PR #46](https://github.com/broadinstitute/catch/pull/46) and documentation is in CATCH's README.

**Supplementary References**

1. Metsky, H. C. et al. Capturing sequence diversity in metagenomes with comprehensive and scalable probe design. *Nature Biotechnology* 37, 160–168 (2019).
2. Alanko, J. N. et al. Syotti: scalable bait design for DNA enrichment. *Bioinformatics* 38, i177–i184 (2022).
3. Wylie, T. N., Wylie, K. M., Herter, B. N. & Storch, G. A. Enhanced virome sequencing using targeted sequence capture. *Genome Research* 25, 1910–1920 (2015).
4. Bonsall, D. et al. ve-SEQ: Robust, unbiased enrichment for streamlined detection and whole-genome sequencing of HCV and other highly diverse pathogens. *F1000Research* 4, 1062 (2015).
5. Paijmans, J. L. A., Fickel, J., Courtiol, A., Hofreiter, M. & Förster, D. W. Impact of enrichment conditions on cross-species capture of fresh and degraded DNA. *Mol. Ecol. Resour.* 16, 42–55 (2016).

| 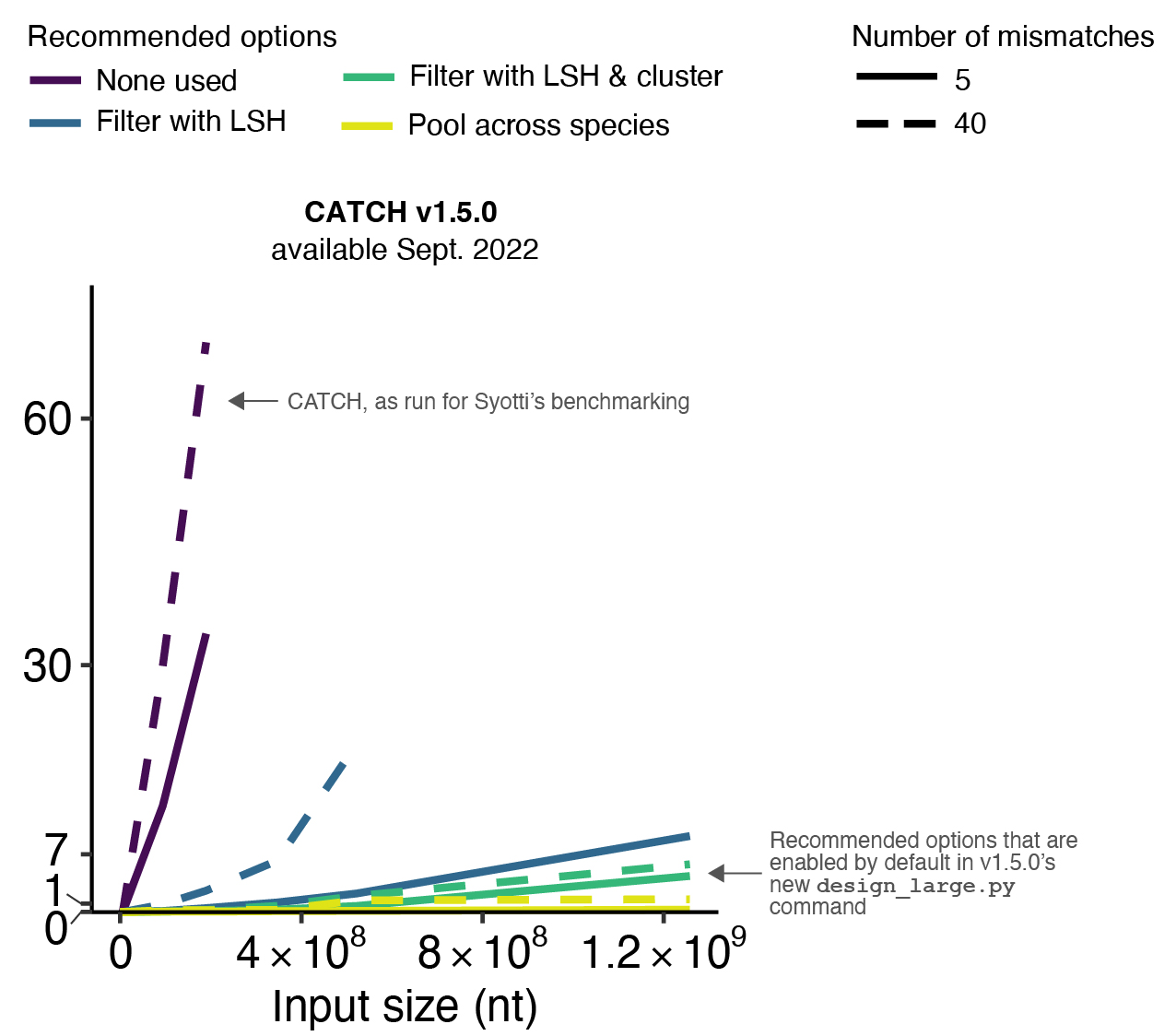 |
| --- |
| **Supplementary Figure 1 — Runtime of CATCH v1.5.0 depends on user-specified arguments.** Same as Figure 1a, except using CATCH v1.5.0, a more recent version available in September 2022. As in Figure 1a, the input is the viral dataset used in Syotti’s benchmarking [2], which we had previously constructed and made available; it contains 588 viral species. Colors indicate a manner of running CATCH: purple indicates using no recommended options, and blue and green indicate using options recommended in CATCH’s publication, README, and help messages. Yellow indicates using viral species labels provided in the data, by handling species independently and pooling probes across them (pooling has little effect on the number of probes owing to low whole-genome homology across species; Supplementary Figure 3). Solid/dashed line types indicate the probe-target divergence (number of mismatches) tolerated in the design; CATCH exhibits lower runtimes at lower values of this parameter, which contrasts with Syotti’s behavior (Figure 1b). Syotti's runtime benchmarking [2] runs CATCH as shown by the dashed purple line, which uses no recommended options and 40 mismatches. The purple lines (no recommended options) are shared with Figure 1a; they were generated using one version of CATCH (v1.4.1) and are equivalent across the two panels because they are computationally expensive to compute and no changes between v1.4.0 and v1.5.0 modify that manner of running CATCH; all other lines are version-dependent. ~1.26×10^9^ nt of input is the full viral dataset; lines that end before that input size represent runs of CATCH that were terminated early owing to their expected runtime. “Filter with LSH” refers to the argument `--filter-with-lsh-minhash 0.6` and “cluster” refers to the arguments `--cluster-and-design-separately 0.15 --cluster-from-fragments 50000`. |

| 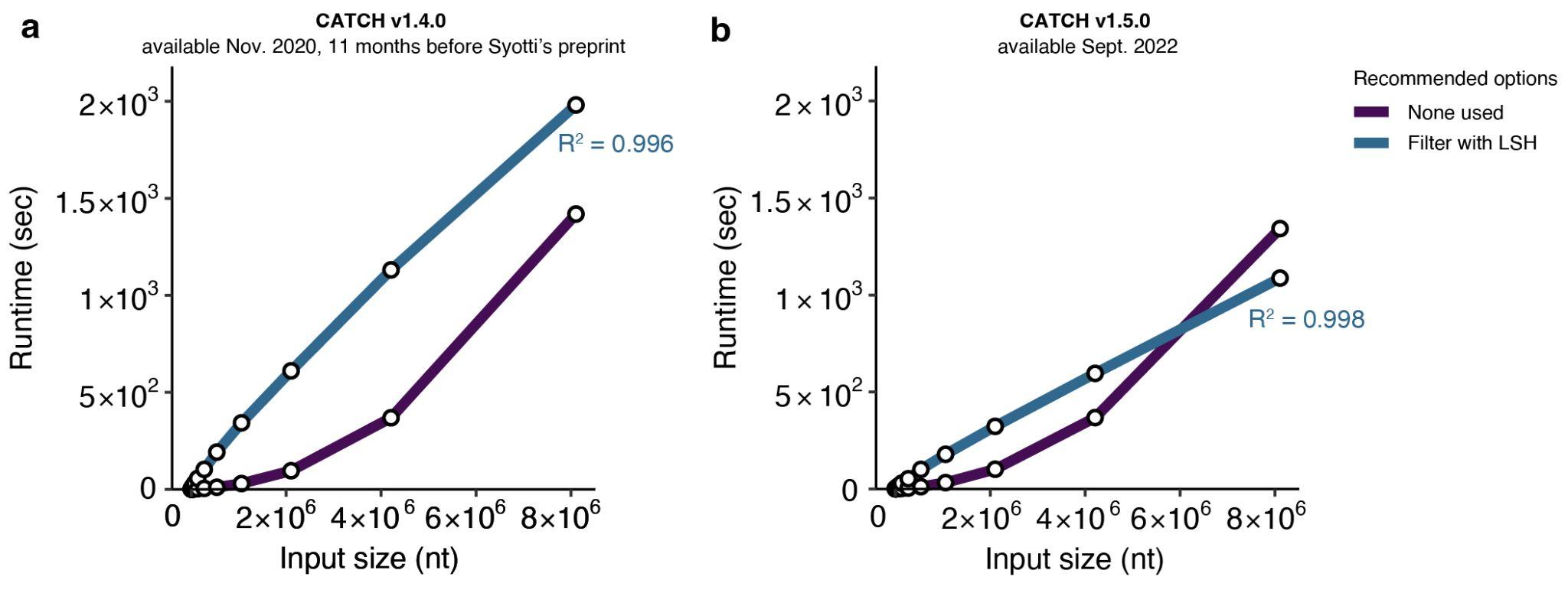 |
| --- |
| **Supplementary Figure 2 — Recommended option improves CATCH’s scalability on the MEGARes dataset.** The runtime of CATCH against increasingly large input samplings. The input is the MEGARes dataset used in Syotti’s benchmarking [2]. Plotted points are measured runtimes, following the sampling strategy in [2], and lines interpolate the points. **a**, Results using CATCH v1.4.0, available 11 months before Syotti was described in a preprint. **b**, Results using CATCH v1.5.0, a more recent version available in September 2022. In both panels, R^2^ evaluates the measurements’ goodness-of-fit to a linear line for the “Filter with LSH” option (blue); that option refers to using the argument `--filter-with-lsh-minhash 0.6`, an option recommended in CATCH’s publication [1], README, and help messages to enhance scalability. As an alternative to the linear blue line, Syotti’s benchmarking [2] runs CATCH as shown by the purple line and therefore reports that CATCH exhibits an upward-bending curve (i.e., super-linear performance) in practice. |

| 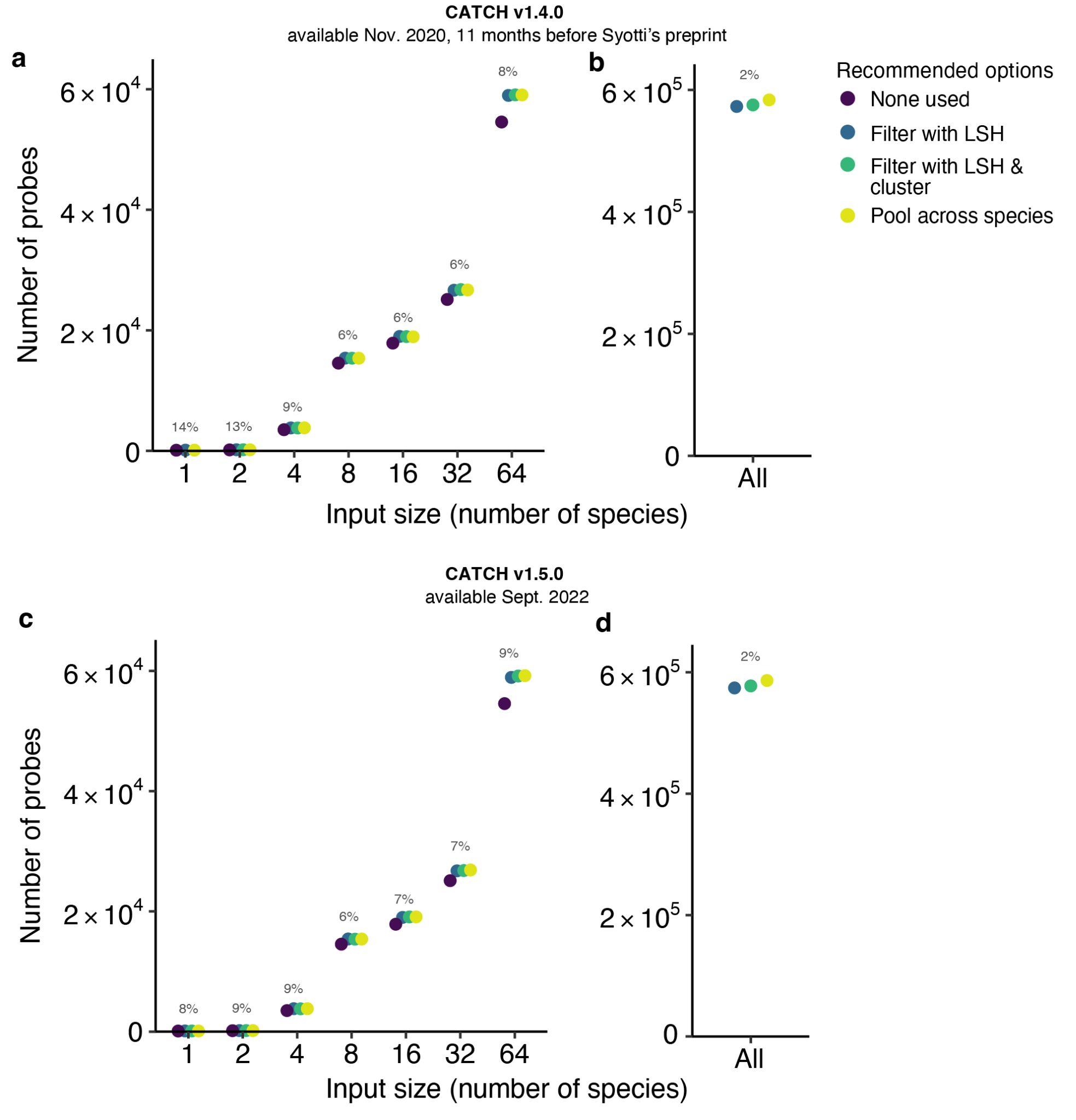 |
| --- |
| **Supplementary Figure 3 — Recommended options lead to a small increase in the number of probes.** The number of probes for different numbers of input species in the viral dataset used in Syotti’s benchmarking, following the same sampling strategy as that benchmarking [2]. Colors indicate manners of running CATCH, as described in Figure 1. **a,** Percents above plotted points are the percent increase in the number of probes between the “None used” (purple) and “Pool across species” (yellow) points. Inputs are up to 64 species because we could not efficiently obtain data for the “None used” data point beyond 64 species. **b,** Input is all viral data, shown on a separate scale from a. Percent above plotted points is the percent increase in the number of probes between the “Filter with LSH” (blue) and “Pool across species” (yellow) points. a and b use CATCH v1.4.0, available 11 months before Syotti was described in a preprint. **c**, same as a except using CATCH v1.5.0. **d**, same as b except using CATCH v1.5.0. The purple dots (no recommended options) were generated using only one version of CATCH and are duplicated across the panels because they are computationally expensive to compute and no changes between v1.4.0 and v1.5.0 affect that manner of running CATCH; all other dots are version-dependent. In a, the green dot is missing at *n*=1 input species because the first species in our sampling has only 1 genome sequence and CATCH v1.4.0, with the corresponding options, crashes due to a bug when given *n*=1 input sequence. |

1. We followed the input sampling strategy in [2], in which entire groups of species are introduced into the input in powers of 2 (1 species, 2 species, 4 species, 8 species, etc.). In our randomized ordering of species, the first 64 species encompass 15.1% of nucleotides in the full dataset. For the manner of running CATCH used in [2] (i.e., no recommended options), in Figure 1a and Supplementary Figure 1, inputs of 128 species and beyond were terminated early owing to their expected runtime; thus, relative performance values are calculated at an input of 64 species (15.1% of nucleotides). [↑](#footnote-ref-2)
2. [2] cites Agilent's advice that 33% divergence is tolerable. While it is likely tolerable, available data suggests it is less than ideal. One study [5] uses Agilent's capture technology and finds enrichment decreasing steadily as divergence rises from 0% to 25%, with preferential enrichment at lower divergences (0–3%) compared to higher divergences (10–25%). The results show some enrichment at 25% divergence but do not contain data past 25%. We are not aware of public studies demonstrating robust enrichment at 33% divergence. It is worth noting our speculation that, since capture panels are almost a commodity, it is in DNA manufacturers’ economic interest to advertise that high divergence values are tolerated by their chemistries. [↑](#footnote-ref-3)
3. For this analysis, [2] downloads pre-designed, publicly available probe sets and cites [1] as the reason for assuming 5 mismatches in the design of those probe sets. That is mistaken: those downloaded probe sets are more recent and are of different sizes and cover more viral targets than the ones detailed in [1], and they use different design parameters (found in params.tsv files [here](https://github.com/broadinstitute/hybsel-design-runs/tree/master/probe-sets/all-human-viruses/2018-10/pooled)). [↑](#footnote-ref-4)
